# *In Vitro* ELISA and Cell-Based Assays Confirm the Low Immunogenicity of VNAR Therapeutic Constructs in a Mouse Model of Human RA: An Encouraging Milestone to Further Clinical Drug Development

**DOI:** 10.1155/2020/7283239

**Published:** 2020-02-03

**Authors:** Obinna C. Ubah, Andrew J. Porter, Caroline J. Barelle

**Affiliations:** ^1^Elasmogen Ltd., Liberty Building, Foresterhill Road, Aberdeen AB25 2ZP, UK; ^2^School of Medical Sciences, Scottish Biologics Facility, University of Aberdeen, Foresterhill Road, Aberdeen AB25 2ZP, UK

## Abstract

Anti-drug antibodies (ADAs), specific for biotherapeutic drugs, are associated with reduced serum drug levels and compromised therapeutic response. The impact of ADA on the bioavailability and clinical efficacy of blockbuster anti-hTNF-*α* monoclonal antibodies is well recognised, especially for adalimumab and infliximab treatments, with the large and complex molecular architecture of classical immunoglobulin antibody drugs, in part, responsible for the immunogenicity seen in patients. The initial aim of this study was to develop solid-phase enzyme-linked immunosorbent assays (ELISA) and an *in vitro* cell-based method to accurately detect ADA and estimate its impact on the preclinical *in vivo* efficacy outcomes of two novel, nonimmunoglobulin VNAR fusion anti-hTNF-*α* biologics (Quad-X™ and D1-NDure™-C4) and Humira®, a brand of adalimumab. Serum drug levels and the presence of ADA were determined in a transgenic mouse model of polyarthritis (Tg197) when Quad-X™ and Humira® were dosed at 1 mg/kg and D1-NDure™-C4 was dosed at 30 mg/kg. The serum levels of the Quad-X™ and D1-NDure™-C4 modalities were consistently high and comparable across all mice within the same treatment groups. In 1 mg/kg and 3 mg/kg Quad-X™- and 30 mg/kg D1-NDure™-C4-treated mice, an average trough drug serum concentration of 8 *μ*g/mL, 50 *μ*g/mL, and 350 *μ*g/mL, respectively, were estimated. In stark contrast, Humira® trough serum concentrations in the 1 mg/kg treatment group ranged from <0.008 *μ*g/mL to 4 *μ*g/mL with trace levels detected in 7 of the 8 animals treated. Trough serum Humira® and Quad-X™ concentrations in 3 mg/kg treatment samples were comparable; however, the functionality of the detected Humira® serum was significantly compromised due to neutralising ADA. The impact of ADA went beyond the simple and rapid clearance of Humira®, as 7/8 serum samples also showed no detectable capacity to neutralise hTNF-*α*-mediated cytotoxicity in a murine fibrosarcoma (L929) cell assay. The neutralisation capacity of all the VNAR constructs remained unchanged at the end of the experimental period (10 weeks). The data presented in this manuscript goes some way to explain the exciting outcomes of the previously published preclinical *in vivo* efficacy data, which showed complete control of disease at Quad-X™ concentrations of 0.5 mg/kg, equivalent to 10x the *in vivo* potency of Humira®. This independent corroboration also validates the robustness and reliability of the assay techniques reported in this current manuscript, and while it comes with the caveat of a mouse study, it does appear to suggest that these particular VNAR constructs, at least, are of low inherent immunogenicity.

## 1. Introduction

Therapeutic monoclonal antibodies have seen great success in the treatment of a wide range of conditions ranging from autoinflammatory diseases to cancers. Despite these major therapeutic milestones, a significant risk of immunogenicity is associated with this class of protein-based therapeutics, with a real threat of therapeutic failure and even severe adverse events particularly with anti-TNF-*α* protein-based therapeutics [1–7]. A number of drug-related factors have been implicated in the initiation of an immunogenic reaction and include but are not limited to the primary sequence of the biotherapeutic drug, formulation changes, aggregation, host-cell specific posttranslational modifications, the presence of host-cell proteins, chemical modifications (deamination, oxidation, or glycation), and changes in protein structure [7–10].

There is overwhelming evidence of an anti-drug antibody (ADA) being generated *in vivo* against a number of clinically important anti-TNF-*α* biologics, and a direct link between this ADA response, serum drug disappearance, and therapeutic failure [1, 3, 10–17]. In one case study, ADA to Humira® (currently the world's bestselling drug, 2018) was detected after three years of treatment in about 28% of 272 Humira®-treated patients, with 67% of those patients developing ADA after just 28 weeks, resulting in exacerbation of disease [18].

There is a clear need, especially for these refractory patients, for a next-generation potent hTNF-*α*-neutralising biologic with inherent low immunogenicity. The structurally less complex biologics delivered from the variable new antigen receptor (VNAR) drug platform, and related humanised formats known as soloMERs™ [19–22], have both previously been reported as having inherently low immunogenicity in a classical dendritic cell-T-cell assay [21]. Their smaller molecular size, simple single-chain format, minimal requirements for posttranslational modification, and excellent stability, may all contribute to this low immunogenicity profile.

Immunogenicity assessment has quite rightly become one of the regulatory cornerstones of biotherapeutic drug approvals. Unfortunately, the simple prediction of immunogenicity and its resulting knock-on clinical significance remains challenging. However, as methodologies (*in silico*, *in vitro*, and possibly *in vivo* models) develop, the ability to more closely predict and mitigate risks of immunogenicity are beginning to improve, but even with these new approaches, one single assay of immunogenicity prediction is probably not achievable. Enzyme-linked immunosorbent assay (ELISA) is the most commonly used assay platform for immunogenicity assessment [1, 23]. ELISA assays can be vulnerable to interference from a range of serum components such as rheumatoid factors or excess drug in trough serum samples, and bridging-type ELISAs (bELISAs) have been shown to have limited capacity to detect long-lived IgG4 ADA species [1, 23–27]. Moreover, and like all solid-phase ELISA techniques, they are sensitive to artefacts such as epitope shielding and neoepitope formation [1, 12]. However, if methods are employed to minimise these risks, then ELISA-based assays are still powerful tools for screening/quantifying drug-ADA immune complexes in biological samples because of their inherent sensitivity, cost/time effectiveness, availability of reagents, assay reproducibility, and flexibility of assay formats. In contrast to ELISA, cell-based assays can be designed to give a functional assessment of a biologically active drug in the presence or absence of ADA (neutralising and non-neutralising). Regulatory authorities recommend that cell-based assays, if available and suitable, are used in concert with other techniques, to quantify neutralising ADA specific for biotherapeutic drugs, and also the impact of ADA on the functionality of a drug candidate [1, 3].

In this manuscript, we describe the application of solid-phase ELISA and classical *in vitro* cell-based approaches to evaluate the presence of an anti-hTNF-*α* drug and ADA in trough mouse serum samples prepared from a transgenic mouse model of human rheumatoid arthritis (RA) disease. Analysis compared the level of ADA production in trough mouse serum samples for two novel anti-hTNF-*α* modalities (Quad-X™ and D1-NDure™-C4) with a clinically available anti-hTNF-*α* monoclonal antibody, Humira®. Furthermore, trough serum drug levels were determined using both direct and indirect ELISA methods, allowing us to better understand and correlate the impact of ADA on the preclinical *in vivo* efficacy study outcomes of these anti-hTNF-*α* drugs [28]. The conventional approach for ADA investigation is to assess serum samples collected at the end of a therapeutic cycle (trough level), where the drug level is low enough to minimise any drug interference in a drug-sensitive assay but not so low as to limit the clinical usefulness of the assay in detecting ADA [1]. Therefore, for the purpose of this report, we refer to the serum samples utilised here as “trough” serum samples, only on the basis that the samples were collected 48 h after the end of a therapeutic cycle.

## 2. Materials and Methods

### 2.1. Protein Drug Formats and Endotoxin Testing

The novel anti-hTNF-*α* VNAR Quad-X™ is a 103 kDa engineered domain with two biparatopic anti-hTNF-*α* VNAR domains (VNAR D1 and C4) fused to the hinge region of a wild-type human IgG1 Fc *via* a short glycine-rich linker, and two additional binding domains (VNAR D1 and C4) C-terminally fused to the CH-3 region of the Fc fragment *via* a longer flexible glycine-rich linker creating a quadrivalent/biparatopic construct harbouring a wild-type human IgG1 Fc fragment. A second anti-hTNF-*α* VNAR drug D1-NDure™-C4 is a 38 kDa linear bispecific construct of the two anti-hTNF-*α* VNAR D1 and C4 domains, respectively, fused *via* flexible glycine-rich linkers to the N- and C-terminal regions of a humanised anti-human serum albumin VNAR, NDure™ [21, 22, 28, 29]. This linear construct incorporates a c-terminal poly-histidine tail and a protein-L binding site in the framework region of the HSA binding partner (NDure™) allowing flexibility for downstream immunodetection and purification. Humira®, also known as adalimumab, is a commercially available 150 kDa recombinant, fully human IgG1 monoclonal antibody specific for hTNF-*α* [30, 31].

All protein drug samples and sterile D-PBS buffer were assessed for endotoxin levels by a LAL (*Limulus amebocyte* lysate) test using the Pierce™ LAL Chromogenic Endotoxin Quantitation Kit (Thermo Fisher Scientific, Rockford, USA). The analysis was performed according to the manufacturer's instructions. All samples were found to have endotoxin levels that were within the limit acceptable for the *in vivo* study (<1.0 EU/mg).

### 2.2. Treated and Untreated Mice Serum Samples

Also described in a recent original research paper [28], three-week-old transgenic Tg197 mice were injected subcutaneously twice weekly for 7 weeks with anti-hTNF-*α* Quad-X™, Humira® at 1 mg/kg or 3 mg/kg, and D1-NDure™-C4 at 30 mg/kg. The vehicle control treatment group received sterile Dulbecco's phosphate-buffered saline (D-PBS) twice weekly for the duration of the treatment. Each treatment group consisted of 8 sex- and age-matched mice (4♂/4♀). Following euthanasia 48 h post final drug administration, blood was drawn from all treated mice *via* cardiac puncture and collected into adequately labelled tubes. After 30 min incubation at room temperature to allow clotting, blood samples were centrifuged for 8 min at 3824 x g (6000 rpm) at 4°C. The supernatants were transferred to clean tubes and further centrifuged for 8 min at 15,294 x g (12,000 rpm) at 4°C. The resulting trough sera supernatants were collected and stored at -80°C prior to the commencement of *in vitro* analysis. Unless otherwise stated, all data presented in this manuscript represents trough sera samples collected from all 8 sex-matched mice per treatment group from a previously reported *in vivo* efficacy study [28].

### 2.3. Direct and Indirect Capture ELISA Detection of Drug and Drug-ADA Complexes

#### 2.3.1. Detecting Anti-TNF-*α* Quad-X™ and Humira® in Treated Mice Sera

MaxiSorp Nunc F96 MicroWell (Thermo Fisher Scientific, Denmark) clear plates were coated with an anti-human IgG (Fc specific) antibody produced in goat (Sigma-Aldrich, USA) using a 1 : 1000 dilution in phosphate-buffered saline (PBS) pH 7.4. Plates were incubated for 1 h at 37°C or overnight (o/n) at 4°C, then washed three times with PBS/0.1% Tween 20 buffer (PBST) before adding 4% (*w*/*v*) milk-PBS (M-PBS) block solution, and incubated for 1 h at 37°C. Plates were washed three times with PBST buffer. As specified previously, PBS-diluted sera samples were added to designated wells (an additional 2-fold dilution). Plates were incubated for 1 h at room temperature before washing three times with PBST. A 1 : 1000 dilution in 4% (*w*/*v*) M-PBST of anti-human IgG- (Fc specific) peroxidase antibody produced in goat (Abcam, UK) was added to the appropriate wells. Plates were further incubated at room temperature for 1 h, before a final wash of 3x PBST and PBS. Colorimetric signal development was performed using 1-Step™ Ultra TMB-ELISA substrate (Thermo Fisher Scientific, USA) and neutralised using 1 M H_2_SO_4_. Colorimetric intensity was determined using a plate reader at 450 nm. For anti-TNF-*α* serum drug quantification, Quad-X™ and Humira® of known concentrations were included in the assay and detected using anti-human IgG- (Fc specific) peroxidase antibody.

#### 2.3.2. Detecting Drug-Mouse ADA (mADA) Immune Complexes in Treated Mice Sera

MaxiSorp Nunc F96 MicroWell clear plates were coated with anti-mouse IgG (Fc specific) antibody produced in goat (Sigma-Aldrich, USA) diluted 1 : 1000 in PBS. All next steps (washes, blocking, and serum sample twofold dilutions across designated wells) were performed as described in Section 2.3.1. A peroxidase-conjugated anti-human IgG (Fc specific) antibody produced in goat or a peroxidase-conjugated anti-poly-histidine antibody produced in rabbit (Abcam, UK), diluted at 1 : 1000 in 4% (*w*/*v*) M-PBST was used for drug-mADA complex detection. Plates were incubated for 1 h at room temperature, then washed 3x with PBST and PBS. Colour development and acid neutralisation were performed as described in Section 2.3.1.

Alternatively, MaxiSorp Nunc F96 MicroWell clear plates were coated with anti-human IgG (Fc specific) antibody produced in goat using a 1 : 1000 dilution in PBS. All next steps were as described in Section 2.3.1. A 1 : 1000 dilution in 4% (*w*/*v*) M-PBST of anti-mouse IgG- (whole molecule) peroxidase antibody produced in sheep (Sigma-Aldrich, USA) was added to designated wells. Plates were incubated for 1 h at room temperature, then washed 3x with PBST and PBS. Colour development and acid neutralisation were performed as described in Section 2.3.1.

#### 2.3.3. Detecting Drug-mADA Complexes in Treated Mice Sera Using Direct Capture ELISA

MaxiSorp Nunc F96 MicroWell clear plates were coated with 1-2 *μ*g/mL recombinant hTNF-*α* (Invitrogen, USA) or HSA (Sigma-Aldrich, USA), and plates were incubated for 1 h at 37°C or o/n at 4°C. All next steps (for D1-NDure™-C4) were as described in Section 2.3.1. A 1 : 1000 dilution of anti-mouse IgG- (whole molecule) peroxidase antibody produced in sheep in 4% (*w*/*v*) M-PBST was added to designated wells. Plates were incubated for 1 h at room temperature and washed 3x with PBST and PBS. Colour development and acid neutralisation were performed as described in Section 2.3.1.

#### 2.3.4. Determining Specificity of the Direct Capture ELISA Using Anti-TNF-*α* Treated Mice Sera

MaxiSorp Nunc F96 MicroWell clear plates were coated with 10 *μ*g/mL human serum albumin (HSA). All proceeding steps involving wash steps, blocking, and sera sample twofold dilutions across designated wells described in Section 2.3.1 were followed. A detection peroxidase-conjugated anti-human IgG (Fc specific) antibody produced in goat or an anti-mouse IgG- (whole molecule) peroxidase antibody produced in sheep was added to designated wells. As a positive control, an anti-human serum albumin soloMER, NDure™ (with a poly-histidine tail) was added at 0.1 *μ*g/mL top concentration and twofold diluted across designated wells. A 1 : 1000 dilution in 4% (*w*/*v*) M-PBST of anti-poly-histidine-peroxidase antibody produced in rabbit was added to designated wells. Plates were incubated for 1 h at room temperature and washed three times with PBST and PBS. Colour development and acid neutralisation were performed as described in Section 2.3.1.

### 2.4. Functional Assessment of Drug in Mice Serum Samples

#### 2.4.1. Direct-Binding ELISA

MaxiSorp Nunc F96 MicroWell clear plates were coated with 1-2 *μ*g/mL recombinant hTNF-*α* or HSA. Coated plates were incubated for 1 h at 37°C or o/n at 4°C. All proceeding steps involving wash steps, blocking, and sera sample twofold dilutions across designated wells described in Section 2.3.1 were followed. A peroxidase-conjugated anti-human IgG (Fc specific) produced in goat or an anti-poly-histidine-peroxidase antibody produced in rabbit was added to designated wells at 1 : 1000 dilution in 4% (*w*/*v*) M-PBST. Plates were incubated for 1 h at room temperature and washed three times with PBST and PBS. Colour development and acid neutralisation were performed as previously described. Functional (nonneutralised) drug in the serum was quantified using known concentrations of D1-NDure™-C4, Quad-X™, and Humira® as standards.

#### 2.4.2. In Vitro L929 Cell-Based Neutralisation Assay

As previously described [22], a TNF-*α*-sensitive mouse fibrosarcoma cell line (L929 cells) was grown to 90% confluence in Dulbecco's Modified Eagle's Medium (DMEM) (Life Technologies Ltd., UK) supplemented with 10% (*v*/*v*) Gibco® heat-inactivated fetal bovine serum (Thermo Fisher Scientific, UK) before seeding 5 × 10^3^ cells/well into CELLSTAR® sterile 96-well cell culture flat-bottom plates, with a lid (Greiner Bio-One, Germany). Seeded cells were incubated for 48 h at 37°C and 5% (*v*/*v*) CO_2_. Known concentrations of the control anti-TNF-*α* D1-NDure™-C4, Quad-X™, and Humira® and estimated concentrations of drugs in serum samples were prepared in fresh DMEM media before adding to designated wells containing L929 cells. Cells were immediately treated with 1 *μ*g/mL actinomycin-D and 0.3 ng/mL hTNF-*α* and incubated for 24 h before adding a cell proliferation reagent WST-1 (Roche Diagnostics, Germany). After an additional 24 h of incubation, cell viability was quantified by measuring absorbance at 450 nm.

### 2.5. Data Analysis

All experiments reported are the blank corrected mean of two independent experiments with two replicates per experiments, unless otherwise stated. GraphPad Prism 8 software was used for graphical presentation and statistical analyses where necessary. Where required, *p* values less than 0.05 were considered as statistically significant. Experimental signal cut-off point (threshold) was determined by analysing ≈64 replicate negative control (D-PBS-treated serum samples) data using the following formula: Mean (negative controls) + 3 SD (negative controls).

## 3. Results

### 3.1. Detection of Serum Anti-TNF-*α* Drugs (Humira® and Quad-X™) and Drug-ADA Complex in Pooled Sera Samples

**Figure 1:**
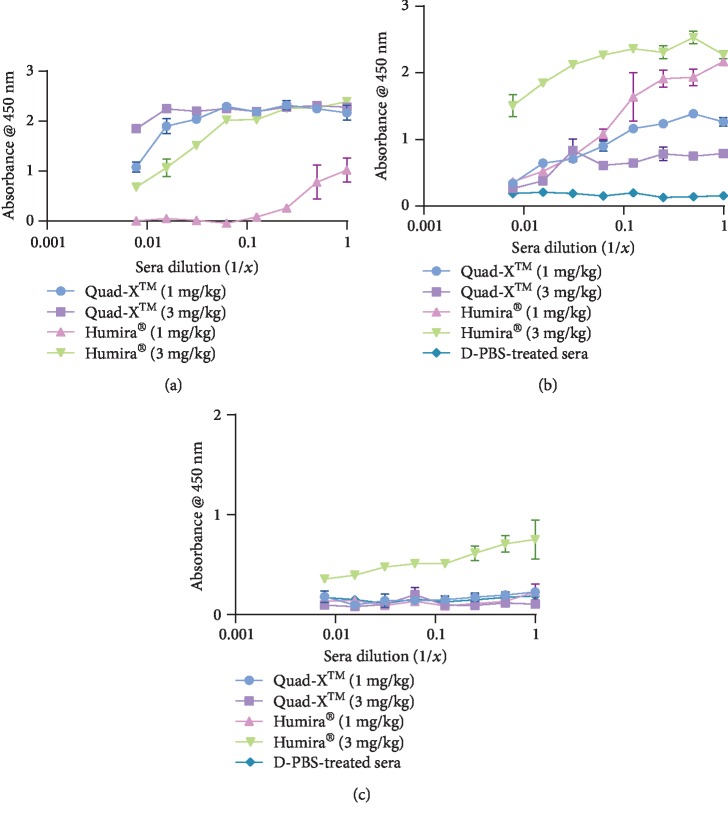
Drug and mouse ADA detection using an indirect capture ELISA. (a) Serum anti-hTNF-*α* Quad-X™ and Humira® were detected using a capture anti-human IgG antibody (Fc specific) and a detection secondary anti-human IgG antibody- (Fc specific) peroxidase. (b) Detection of mouse ADA to anti-hTNF-*α* Quad-X™ and Humira® using a capture anti-human IgG antibody (Fc specific) and detection with an anti-mouse IgG- (whole molecule) peroxidase. (c) Detection of anti-hTNF-*α* Quad-X™ and Humira® to drug-specific mouse IgG using a capture anti-mouse IgG antibody (Fc specific) and a detection antibody, an anti-human IgG antibody- (Fc specific) peroxidase. All serum samples were randomly pooled and diluted at 1 : 20 in sterile PBS, pH 7.4. The results shown are the mean ± SD (*n* = 2 with two replicates per experiment).

In a preliminary investigation, using indirect capture ELISAs, the presence of anti-hTNF-*α* drugs and drug-mouse ADA complex was determined in three randomly selected serum samples per treatment group. For this initial evaluation, and as this particular technique is unable to distinguish between functional and nonfunctional proteins, equal volumes of these selected samples were pooled before diluting 1 : 20 in sterile PBS and adding to ELISA plates coated with either anti-human IgG (Fc specific) or anti-mouse IgG (Fc specific) antibody. The sera Humira® and Quad-X™ were captured using an anti-human IgG (Fc specific) antibody and detected with an anti-human IgG- (Fc specific) peroxidase antibody (Figure 1(a)). Both 1 and 3 mg/kg Quad-X™ samples showed consistently high serum levels of Quad-X™ drug either as free circulating or in complex with mADA. Only the 3 mg/kg Humira® sample showed a comparable serum level of functional or nonfunctional Humira® drug. Mouse IgG (ADA) to anti-TNF-*α* drug was detected in 1 mg/kg or 3 mg/kg Quad-X™ and Humira® samples by indirectly capturing the anti-TNF-*α* drugs with an anti-human IgG (Fc specific) antibody, and detecting with anti-mouse IgG- (whole molecule) peroxidase (Figure 1(b)), but only the 3 mg/kg Humira® pooled sample showed the measurable presence of mouse ADA-bound drug using a different indirect capture approach (Figure 1(c)). However, the level of detectable mouse ADA to drug (neutralising and/or nonneutralising) was consistently higher in all Humira® serum samples (Figure 1(b)). Anti-TNF-*α* drugs, in complex to mouse ADA, were identified by capturing with anti-mouse IgG (Fc specific) antibody and detecting Humira® and Quad-X™ in pooled serum using an anti-human IgG- (Fc specific) peroxidase antibody (Figure 1(c)). Although only the 3 mg/kg Humira® sample showed significant levels of drug-ADA complex, this indirect capture ELISA relies on capturing circulating mouse IgG (drug and nondrug bound). The detection, therefore, of low or no drug-mouse ADA complex could mean that either there is no ADA bound to the drug, or that free circulating serum mouse IgG preferentially occupied the capture anti-mouse IgG antibody, especially if levels of circulating drug-mouse ADA are depleted as a result of drug clearance mediated by ADA activity. Similarly, in the 10 mg/kg Humira®-treated serum samples, mouse ADA was detected in 4 out of 8 serum samples of Humira® samples using the approach described in Figure 1(b), and none was detected using the Figure 1(c) approach (data not shown). No mouse ADA was detected in 10 mg/kg Quad-X™-treated samples using both methods (data not shown).

### 3.2. Detection of Functional Serum Anti-TNF-*α* Drugs and Nonneutralising ADA in Pooled Sera Samples

**Figure 2:**
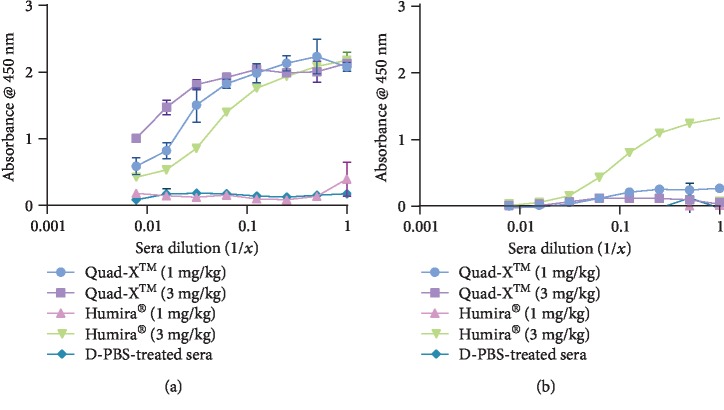
Direct and indirect-binding ELISA for detecting functional serum anti-TNF-*α* drugs and nonneutralising mouse ADA. (a) Direct-binding ELISA of serum samples to 2 *μ*g/mL hTNF-*α* coated wells, with functional drug binding determined using an anti-human IgG antibody- (Fc specific) peroxidase. (b) Indirect-binding ELISA capturing nonneutralising mouse ADA to anti-hTNF-*α* drugs. ELISA wells are coated with 2 *μ*g/mL hTNF-*α*, and nonneutralising mouse ADA was detected using an anti-mouse IgG antibody- (whole molecule) peroxidase. All serum samples were randomly pooled and diluted at 1 : 20 in sterile PBS, pH 7.4. The results shown are the mean ± SD (*n* = 2 with two replicates per experiment).

Figures 2(a) and 2(b) describe a more definitive assessment of anti-TNF-*α* drug functionality and nonneutralising ADA, respectively. In a direct-binding ELISA, serum samples were added to plates coated with hTNF-*α* and functional drug was detected with anti-human IgG- (Fc specific) peroxidase antibody (Figure 2(a)), while nonneutralising mouse ADA was detected with an anti-mouse IgG- (whole molecule) peroxidase antibody (Figure 2(b)). Except for the 1 mg/kg Humira® pooled serum sample, significant levels of functional drugs were detected in the serum samples of 1 mg/kg and 3 mg/kg Quad-X™ and 3 mg/kg Humira® (Figure 2(a)). The presence of potentially nonneutralising ADA bound to a drug was detected only in the Humira® 3 mg/kg pooled samples (Figure 2(b)). This assay reinforces the rapid functional drug clearance seen with 1 mg/kg Humira® samples and suggests that the 3 mg/kg Humira® may have been cleared more quickly than Quad-X™ samples. This assumption is based on the minimal levels of nonneutralising mouse ADA in complex with functional drug in both the 1 mg/kg and 3 mg/kg serum Quad-X™ samples.

### 3.3. Detection of Serum Anti-TNF-*α* Drugs (Humira® and Quad-X™) and Drug-ADA Complex in Individual Mouse Serum Samples

**Figure 3:**
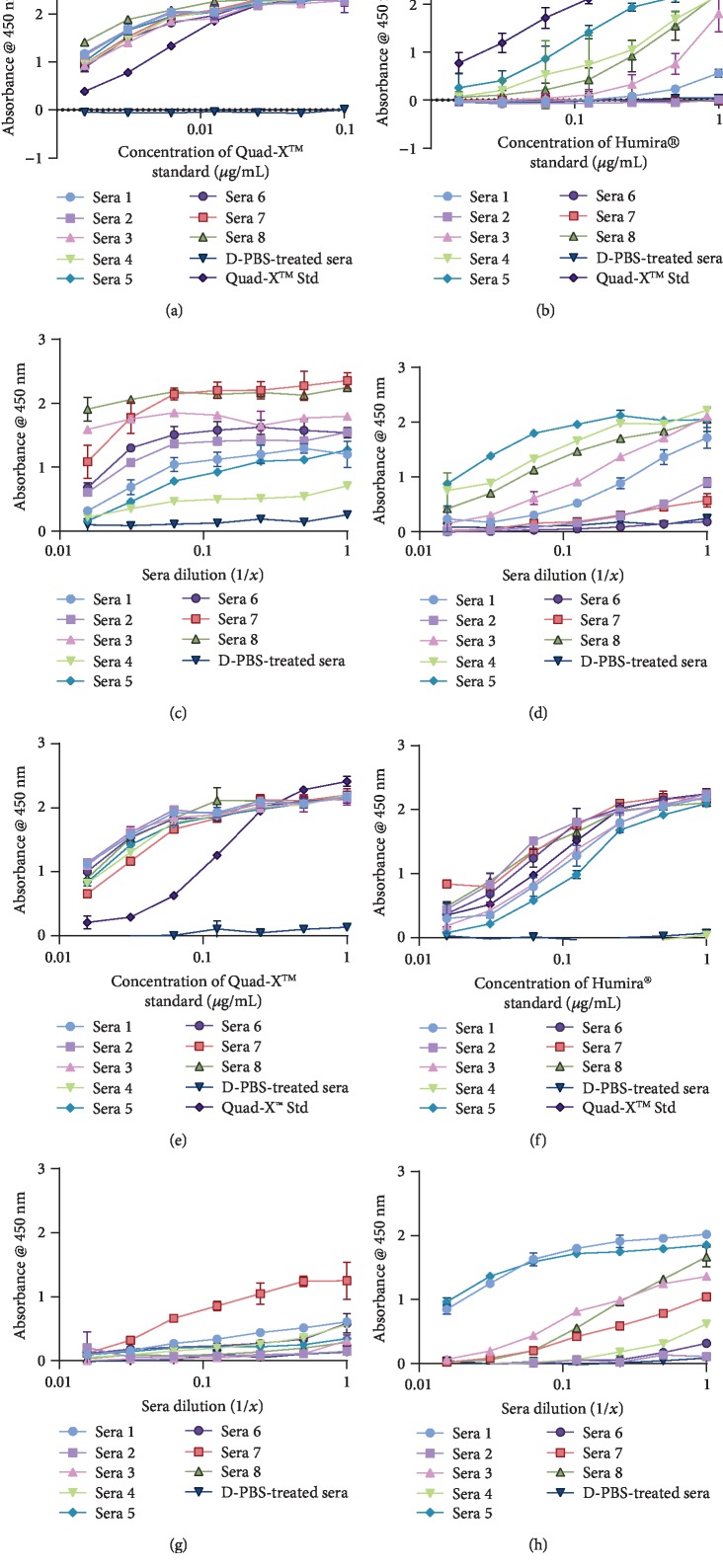
Indirect capture ELISA determination of anti-hTNF-*α* drugs and drug-mouse ADA complexes in individual mouse serum samples. (a and e) Detecting and quantifying serum Quad-X™ in 1 mg/kg and 3 mg/kg treated mice serum samples diluted at 1 : 40 and 1 : 100 in sterile PBS, pH 7.4, respectively, prior to adding to designated ELISA wells. Standard anti-hTNF-*α* Quad-X™ (Quad-X™ Std) was used at a top known concentration of 0.1 *μ*g/mL. (b and f) Detecting and quantifying serum Humira® in 1 mg/kg and 3 mg/kg treated mice serum samples diluted at 1 : 20 and 1 : 100 in sterile PBS, pH 7.4, respectively, prior to adding to designated ELISA wells. Standard Humira® (Humira® Std) was used at a top known concentration of 1 *μ*g/mL. ELISA wells were coated with an anti-human IgG antibody (Fc specific), and detection was performed with an anti-human IgG antibody- (Fc specific) peroxidase. (c and g) Indirect ELISA detection of anti-Quad-X™ mouse ADA to Quad-X™ in 1 mg/kg and 3 mg/kg Quad-X™-treated serum samples diluted at 1 : 20 and 1 : 50 in sterile PBS, pH 7.4, respectively, prior to adding to designated ELISA wells. (d and h) Indirect ELISA detection of a mouse ADA complex to serum Humira® in 1 mg/kg and 3 mg/kg Humira®-treated serum samples diluted at 1 : 20 and 1 : 50 in sterile PBS, pH 7.4, respectively, prior to adding to designated ELISA wells. ELISA wells were coated with an anti-human IgG (Fc specific) antibody, while detection of a drug-ADA complex was performed using an anti-mouse IgG antibody- (whole molecule) peroxidase. The results shown are the mean ± SD (*n* = 2 with two replicates per experiment).

Anti-TNF-*α* drugs in 1 and 3 mg/kg treated serum samples were detected and quantified using a known concentration of the drug in PBS buffer as standard, thereby allowing approximate estimation of the trough amount of total circulating anti-TNF-*α* drug in each mouse serum (Figures 3(a), 3(b), 3(e), and 3(f)). The serum levels of Quad-X™ in both 1 and 3 mg/kg treated samples showed consistent and significantly higher circulating drug concentrations in all treated mice when compared to mice sera from 1 or 3 mg/kg Humira® treatments (Figures 3(a), 3(b), 3(e), and 3(f)). Trough serum drug concentrations in the 1 and 3 mg/kg treated Quad-X™ serum samples were on average 8 *μ*g/mL and 50 *μ*g/mL, respectively, while the trough drug serum levels in the 1 mg/kg Humira®, where a signal was detectable, ranged from <0.008 *μ*g/mL (serum samples 2, 6, and 7—assay LoD) up to 20 *μ*g/mL (serum sample 5), with an average of 4 *μ*g/mL drug in serum samples 1, 3, 4, and 8. This observation is reinforced by further analysis of serum samples 2, 6, and 7 (Figure 3(d)) where trace levels of Humira®-ADA were detected. The levels of drug-mouse ADA complex in the 1 mg/kg Quad-X™ or Humira® sera were quite variable across individual samples (Figure 3(c)). For the 3 mg/kg Humira®-treated samples, an average trough serum drug level of 70 *μ*g/mL was determined (Figure 3(f)). While there is again some variation in data sets, the drug-ADA levels in the 3 mg/kg Humira® treatment group (Figure 3(h)) are higher across some of the serum samples when compared to 3 mg/kg Quad-X™ (Figure 3(g)).

### 3.4. Detection of Serum Anti-TNF-*α* D1-NDure™-C4 and Drug-ADA Complex Levels

**Figure 4:**
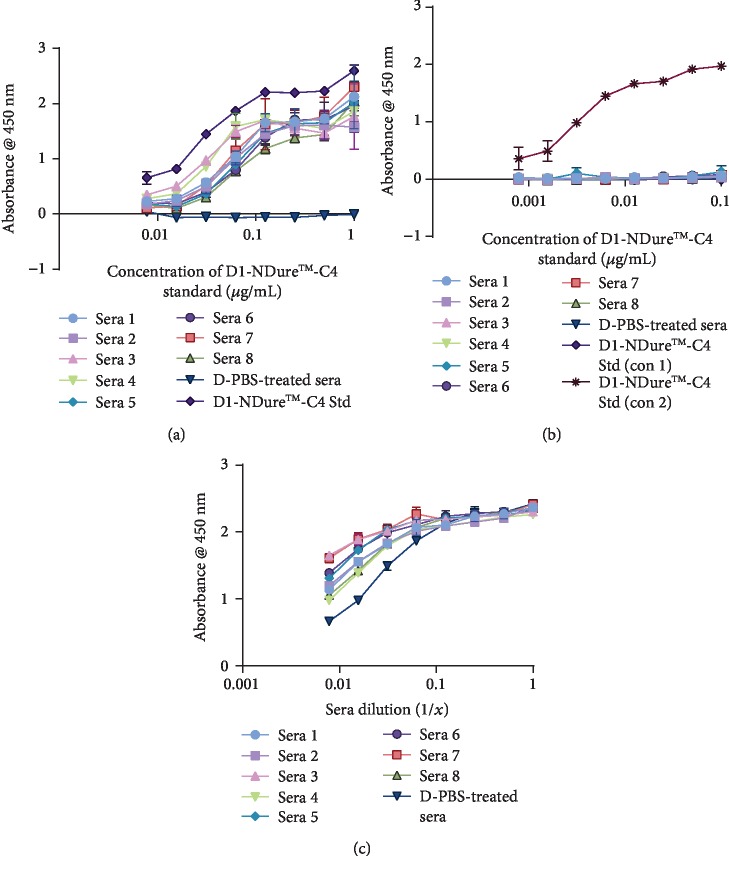
Detection and quantification of serum D1-NDure™-C4 and drug-specific mouse ADA using direct-binding ELISA. (a) Detecting and quantifying serum D1-NDure™-C4 in 30 mg/kg D1-NDure™-C4-treated mice serum samples diluted at 1 : 500 in sterile PBS, pH 7.4 prior to adding to designated ELISA wells. Serum D1-NDure™-C4 was detected and quantified in a direct-binding ELISA with wells coated with 2 *μ*g/mL hTNF-*α*, and secondary antibody detection was performed using an anti-poly-histidine-peroxidase antibody. Standard D1-NDure™-C4 (D1-NDure™-C4 Std) was used at a top known concentration of 1 *μ*g/mL. (b) Direct-binding ELISA detection of mouse ADA to anti-hTNF-*α* drug in 30 mg/kg D1-NDure™-C4 serum samples diluted at 1 : 100 in sterile PBS, pH 7.4 prior to adding to designated ELISA wells. Detection of drug-specific mouse ADA was determined using wells coated with 2 *μ*g/mL hTNF-*α* and an anti-mouse IgG antibody- (whole molecule) peroxidase as detection reagent. D1-NDure™-C4 Std (con 1) and D1-NDure™-C4 Std (con 2) are controls used at known concentrations, with secondary antibody detection using an anti-mouse IgG antibody- (whole molecule) peroxidase or an anti-poly-histidine-peroxidase antibody, respectively. The results shown are the mean ± SD (*n* = 2 with two replicates per experiment). (c) Detection of circulating serum mouse IgG in 30 mg/kg D1-NDure™-C4-treated mice serum samples diluted at 1 : 500 in sterile PBS, pH 7.4 prior to adding to designated ELISA wells. Indirect capture ELISA wells coated with an anti-mouse IgG (Fc specific) antibody and serum mouse IgG detected using an anti-mouse IgG antibody- (whole molecule) peroxidase were used. The result shown are the mean ± SD (*n* = 1 with two replicates per experiment).

Following multiple binding curve calibrations used to test different initial serum dilution factors of 30 mg/kg D1-NDure™-C4 serum samples (data not shown), an initial serum sample dilution factor of 1 : 500 in sterile PBS produced a typical sigmoidal drug-binding curve. Circulating serum D1-NDure™-C4 was calculated using a known concentration of D1-NDure™-C4 recombinant protein as a control. Using this approach, an average trough serum level of functional free circulating and/or nonneutralising ADA-complexed D1-NDure™-C4 was calculated to be 350 *μ*g/mL. Functional D1-NDure™-C4 in sera was determined in an hTNF-*α*-coated direct-binding ELISA, with hTNF-*α* binding detected using an anti-poly-histidine-peroxidase antibody (Figure 4(a)). Both the direct (Figure 4(b)) and indirect capture ELISA (data not shown) formats used here to reveal mouse ADA to D1-NDure™-C4 drug consistently showed no measurable D1-NDure™-C4-specific ADA in sera. In a previous sensitivity/limit of detection assay, we established a limit of detection (LoD) for total mouse IgG in mouse serum samples at ≈1 ng/mL. Free circulating mouse IgG in both D1-NDure™-C4- and D-PBS-treated mice serum samples were detected using an indirect capture ELISA (Figure 4(c)) demonstrating the robustness of the assay to detect circulating mouse IgG. This data shows comparable levels of circulating mouse IgG in both drug- and D-PBS-treated samples, therefore supporting the observations that the titre of serum D1-NDure™-C4-specific mouse ADA is either present at trace levels (below <1 ng/mL) or that the drug did not elicit any mouse ADA. While unlikely, one alternative interpretation of the data is that drug interference is due to the high *in vivo* dosing regimen of D1-NDure™-C4 (30 mg/kg). As mentioned earlier, we succeeded in detecting mouse ADA in complex with Humira® in 10 mg/kg treated serum samples with an initial serum sample dilution of 1 : 100, although drug interference may have limited the capacity of the assay to detect ADA in all serum samples (data not shown).

### 3.5. Assessing *In Vitro* Functionality of Anti-TNF-*α* Drug in Serum Samples

**Figure 5:**
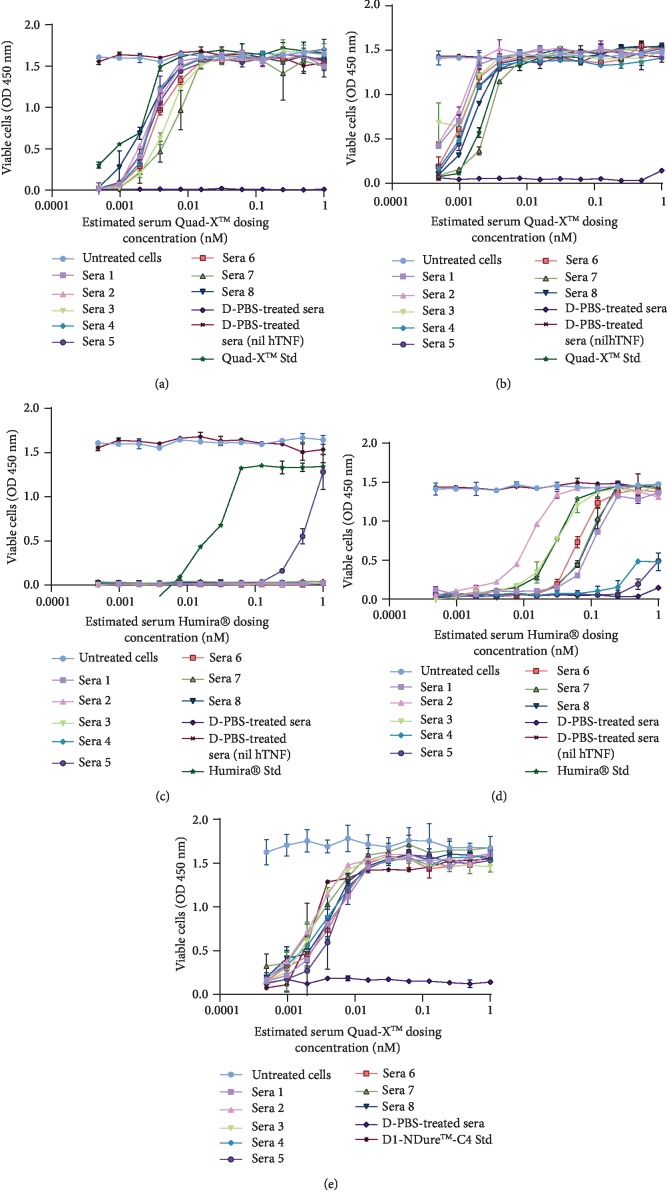
In vitro functional assessment of serum samples of mice treated with anti-hTNF-*α* drugs. (a–e) Determination of residual neutralising capacity of serum anti-hTNF-*α* drugs in a classical *in vitro* hTNF-*α*-induced cytotoxicity assay using a sensitised L929 cell line. (a and b) Shown are 1 mg/kg and 3 mg/kg Quad-X™-treated serum samples, respectively (Quad-X™-treated sera vs. D-PBS-treated sera, *p* < 0.0001). (c and d) Shown are 1 mg/kg and 3 mg/kg Humira®-treated serum samples, respectively (Humira®-treated sera vs. D-PBS-treated sera, *p* = 0.9991 for all 1 mg/kg treated sera except sera 5 (*p* < 0.001) and *p* > 0.7 for 3 mg/kg treated sera 4 and 5). (e) Shown are 30 mg/kg D1-NDure™-C4-treated serum samples (*p* < 0.0001 for all D1-NDure™-C4-treated sera vs. D-PBS-treated sera). Exogenous recombinant hTNF-*α* at 0.3 ng/mL and 1 *μ*g/mL actinomycin-D were used in all treatment groups except where otherwise stated. No exogenous hTNF-*α* and actinomycin-D were added to untreated cells and D-PBS-treated sera (nil hTNF) control groups. Quad-X™ Std and Humira® Std represent standard known concentrations of the drugs. The results shown are the mean ± SD (*n* = 2 with three replicates per experiment). Results were analysed statistically using a two-way ANOVA with multiple comparison post hoc test (*p* > 0.05 is considered not significant).

The capacity of circulating serum anti-hTNF-*α* drugs to block exogenous hTNF-*α*-mediated cytotoxicity in a sensitised fibrosarcoma cell line (L929) was assessed in vitro. Both 1 mg/kg and 3 mg/kg Quad-X™ serum samples showed no apparent loss of activity with comparable hTNF-*α*-neutralising potency to “standard” Quad-X™ recombinant protein (Figures 5(a) and 5(b)). Serum samples from the second VNAR format, D1-NDure™-C4 serum also demonstrated comparable potency to control protein in the L929 cell assay (Figure 5(e)). For the 1 mg/kg Humira®-treated mice serum samples, only one serum sample [5] showed any (and weak) hTNF-*α*-neutralising activity (Figure 5(c)). Although the 3 mg/kg Humira® samples retained more activity compared to the 1 mg/kg treatment group, only traces of activity were recoverable from samples 4 and 5 and only samples 2 and 3 had a potency equivalent to that of the standard clinical control drug (Figure 5(d)). All cells treated with D-PBS-treated serum controls, without the addition of exogenous hTNF-*α*, showed cell viabilities comparable to the untreated control cells (Figures 5(a)–5(d)). This absence of cytotoxicity in the D-PBS-treated serum samples is because the trough serum level of endogenously overexpressed hTNF-*α* in this transgenic disease model (Tg197) is estimated to be significantly below the LD_50_ level (0.25 ng/mL) required for an induction of cytotoxicity in the sensitised L929 cells. It is known that circulating hTNF-*α* levels in Tg197 without treatment, peaks at week 3 (0.45 ng/mL), followed by a consistent decline to 0.22 ng/mL at week 6, and a further steady drop-off in concentration until week 16 [32]. Therefore, this additional experimental control confirms the absence of experimental artefacts or interference from endogenous hTNF in the serum samples tested in this study.

### 3.6. Detection of Nonspecific Binding Signals in Quad-X™ and Humira® Serum Samples

**Figure 6:**
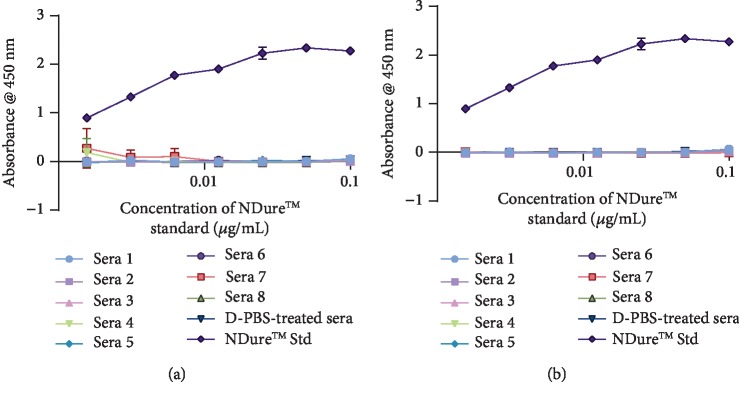
Detection of nonspecific background signals in drug- and D-PBS-treated serum samples. (a–b) Detection of a nonspecific binding response from 1 mg/kg Quad-X™- and Humira®-treated mouse serum samples, respectively. In a direct-binding ELISA, 2 *μ*g/mL human serum albumin (HSA) was used as coating antigen and nonspecific binding was measured using an anti-mouse IgG- (whole molecule) peroxidase antibody. The results shown are the mean ± SD (*n* = 2 with three replicates per experiment, except for the pooled NDure™ Std data which is an *n* = 4 with two replicates per experiment). *p* > 0.99 for all Quad-X- and Humira-treated serum samples vs. D-PBS-treated serum samples, *p* < 0.0001 for NDure™-treated vs. D-PBS-treated serum samples. Results were analysed statistically using a two-way ANOVA with multiple comparison post hoc test (*p* > 0.05 is considered not significant).

A final confirmatory assay was performed to eliminate any possibility of nonspecific binding signals being detected with either the anti-mouse IgG- (whole molecule) peroxidase or anti-human IgG- (Fc specific) peroxidase antibody. Using a direct-binding ELISA and HSA as an unrelated coating antigen (Figures 6(a) and 6(b)), neither the 1 mg/kg Quad-X™, 1 mg/kg Humira® nor D-PBS serum samples (or other unspecified components in the serum samples) showed any interference with or nonspecific binding to the coated HSA. A similar background interference assessment was conducted for 3 mg/kg Quad-X™ and Humira® serum samples with a measured absorbance identical to that of blank controls (data not shown).

## 4. Discussion

The link between ADA, decreased serum drug concentration, and clinical efficacy of genetically engineered immunoglobulin-based anti-hTNF-*α* biopharmaceuticals is very well established [3, 13–17]. A number of studies have followed the immunogenicity of anti-hTNF-*α* monoclonal antibodies in patients and reported a strong effect on treatment response in several conditions including RA, inflammatory bowel disease, psoriatic arthritis, and psoriasis. In each case, the presence of ADA was associated with low to absent serum drug levels, a weakened therapeutic response, and even an exacerbation of the underlying disease [6, 18, 33–37]. The immunogenicity of Humira® has been described throughout the drug's historic approval process, with initial reports of mouse anti-Humira® antibody (MAHA) and later primate anti-Humira® antibody (PAHA). Neutralising ADA was measurable even after a single dose and was reported to adversely affect drug elimination and potency [38].

We have previously reported superior *in vitro* and *in vivo* efficacy of the anti-TNF-*α* Quad-X™ over Humira® and have shown that much of this 10x improvement in potency is due to the empirical design of the Quad-X™ format [22, 28]. The low inherent immunogenicity of the VNAR domains and its humanised soloMER™ derivatives has been previously confirmed experimentally [21]. Here, the serum levels of Humira® in treated mice were lower compared with the Quad-X™-treated samples, with the 1 mg/kg Humira® group showing a severe depletion in drug levels in the pooled serum samples (Figure 1(a) and 2(a)). In addition, the functional anti-hTNF-*α* drug is absent in the 1 mg/kg Humira®-treated samples with this data further supporting the reported outcome of a preclinical *in vivo* efficacy study where 1 mg/kg Humira® treatment was ineffective at blocking disease progression in Tg197 mice, but complete control was achieved by Quad-X™ at 0.5 mg/kg [28]. Assessing drug levels in unmasked individual serum samples further reinforced the evidence of anti-Humira® mouse ADA-mediated rapid clearance of Humira® from the systemic circulation of the treated mice (Figures 3(a)–3(f)). Even the higher levels of Humira® seen in the 3 mg/kg treated serum samples (Figure 3(f)) was only 10% of the corresponding Quad-X™ levels (Figure 3(e)) and would support the partial disease control seen *in vivo* with the 3 mg/kg Humira® dose [28]. The levels of mouse ADA in complex with drugs seen in 1 mg/kg Quad-X™-treated samples were comparable to that seen in 1 mg/kg Humira® samples (Figures 3(c) and 3(d), respectively); however, that level was significantly lower in 3 mg/kg Quad-X™ compared to the corresponding Humira®-treated samples (Figures 3(g) and 3(h), respectively).

One plausible explanation for the no drug-ADA complex detection in some samples in Figure 1(c) could be the effect of steric hindrance caused by excessive mouse ADA bound to the Fc portion of the drug, thereby blocking the binding of the anti-human IgG- (Fc specific) peroxidase antibody used to detect drug-bound mouse IgG (false negative outcome). Since the capture antibody is not selective for the mouse IgG in complex with drug, it is also conceivable that competition for binding to the capture antibody will persist between free circulating mouse IgG and the drug-mouse IgG complex. This competition will favour free circulating mouse IgG if the level of drug-mouse ADA complex is low, due to drug clearance for instance, or to the inherent low immunogenicity of the drug (e.g., Quad-X™ samples). For Humira® samples, it seems more appropriate (when considering the other assay outcomes) to assume that the negligible levels of mouse ADA bound to drug captured in the 1 mg/kg pooled serum samples (Figure 1(c)) was due to ADA-mediated rapid drug clearance. However, even if this assumption is correct, this potentially contradictory data does reinforce the fact that these indirect capture ELISA assays present some challenges when seeking the “best” analytical approach. We believe our thorough interrogation of samples, using more than one assay design to test the same proposition (Figures 1(b) and 1(c)), limits the impact of false positive or negative outcomes.

The immunogenicity of VNARs and humanised VNARs (soloMER™) as drug candidates has previously been assessed in a T-cell proliferation assay, and both developed very low response index (RI) values [21]. Therefore, the soloMER domain (NDure™) used in this study, which is fully cross-reactive with both mouse and human serum albumin [19, 35], was expected to extend the serum half-life of the linear VNAR fusion protein without encouraging a MAHA response. This expectation was confirmed by analysis as the level of functional serum drug was consistently high across all serum samples tested (Figure 4(a)). Furthermore, using the same direct functional binding ELISA technique, a drug-mouse ADA complex was undetectable in all serum samples (Figure 4(b)). The confidence in this is further reinforced in Figure 4(c) where a uniformly high level of free circulating mouse IgG was detected in all test samples. The assay LoD for circulating mouse IgG was approximately 1 ng/mL, while 8–10 ng/mL was obtained as the LoD for serum D1-NDure™-C4, Quad-X™, and Humira® (data not shown). These figures are similar to other published studies [34] and reinforce again the sensitivity and reliability of the assays used. One additional benefit of this high sensitivity is the accommodation (without loss of signal) of significant dilution of trough serum samples, thereby limiting potential assay interferences from serum anti-hTNF-*α* drugs or other uncharacterised mouse serum components.

Regulatory authorities recommend, where possible, the use of cell-based assays to quantify the effect of neutralising ADA against biotherapeutic drugs [1, 3, 7, 39]. Cell-based immunogenicity assessment assays are typically considered to be difficult to standardise, have low sensitivity, are time consuming to perform, require a cell culture facility, and are particularly prone to serum matrix interference [1, 8, 39]. However, cell-based assays are considered much more reliable when assessing the functionality of serum drugs and the effect, if any, of ADA on drug function. Neutralisation of hTNF-*α*-induced cytotoxicity in sensitised L929 cells remains one of the gold-standard assays for the *in vitro* assessment of potency for all anti-hTNF-*α* biologics. The L929 assay is known for its high sensitivity and specificity. The functionality of serum Quad-X™ (Figures 5(a) and 5(b)) and D1-NDure™-C4 (Figure 5(e)) in L929 assays was consistent and statistically significant across all serum samples, and equivalent to the recombinant protein controls. The 1 and 3 mg/kg Humira® serum samples (Figures 5(c) and 5(d), respectively) showed a significant loss of drug function in the majority of serum samples, with the 1 mg/kg Humira® samples showing an almost complete absence of activity. Both the anti-hTNF-*α* Quad-X™ and Humira® retain the Fc portion of the human IgG1 molecule; therefore, the level of recruitment of anti-allotypic mouse ADA to this particular region would be expected to be comparable. However, the Quad-X™ may be protective through “top-and-tail” steric hindrance introduced by the quadrivalent format which places binding sites at both the N and C terminus of the Fc region, possibly limiting access for anti-allotypic ADA binders.

In summary, the cell-based functional assays confirm and mirror the ELISA-based immunogenicity assays for all treatment groups and together this data supports previous *in vivo* efficacy data [26].

The primary objectives of this communication were to present robust and reproducible assay techniques for assessing the functional state of serum VNAR/soloMER™-based biotherapeutic drugs, and the presence and impact of anti-drug antibodies in (nonclinical) serum samples. Animal models, particularly transgenic [37–40], are increasingly being used to study the immunogenicity of therapeutic proteins, with data generated considered as a predictive guide to different aspects of immunogenicity assessment during later drug development. Here, both anti-hTNF-*α* Quad-X™ and D1-NDure™-C4 consistently showed no impact on functionality in the presence or absence of nonneutralising mouse ADA. The previously reported and superior preclinical efficacy and now the apparent low immunogenicity of drug leads support the continued clinical development of the anti-TNF-*α* Quad-X™ and D1-NDure™-C4, with *in vivo* preclinical pharmacokinetic, toxicology, and soloMERisation (humanisation) of both formats planned as the final development steps to clinical candidates. This low inherent immunogenicity is in stark contrast to Humira® which has been dogged by ADA issues throughout its path to clinical approval and subsequent clinical use, with approximately one in three patients becoming refractory to extended therapy [16, 40–42].

## Data Availability

The authors confirm that the data supporting the findings of this study are available within the article, and raw data as generated from our laboratory supporting these findings are available from the corresponding author on request.
